# Semiquantitative RT-PCR measurement of gene expression in rat tissues including a correction for varying cell size and number

**DOI:** 10.1186/1743-7075-4-26

**Published:** 2007-11-26

**Authors:** Maria del Mar Romero, Maria del Mar Grasa, Montserrat Esteve, José Antonio Fernández-López, Marià Alemany

**Affiliations:** 1 Department of Nutrition and Food Science, Faculty of Biology, University of Barcelona, Barcelona, Spain; 2 Ciber Fisiopatologia Obesidad y Nutrición (CB06/03) Instituto de Salud Carlos III, Madrid, Spain

## Abstract

**Background:**

Current methodology of gene expression analysis limits the possibilities of comparison between cells/tissues of organs in which cell size and/or number changes as a consequence of the study (e.g. starvation). A method relating the abundance of specific mRNA copies per cell may allow direct comparison or different organs and/or changing physiological conditions.

**Methods:**

With a number of selected genes, we analysed the relationship of the number of bases and the fluorescence recorded at a present level using cDNA standards. A lineal relationship was found between the final number of bases and the length of the transcript. The constants of this equation and those of the relationship between fluorescence and number of bases in cDNA were determined and a general equation linking the length of the transcript and the initial number of copies of mRNA was deduced for a given pre-established fluorescence setting. This allowed the calculation of the concentration of the corresponding mRNAs per g of tissue. The inclusion of tissue RNA and the DNA content per cell, allowed the calculation of the mRNA copies per cell.

**Results:**

The application of this procedure to six genes: Arbp, cyclophilin, ChREBP, T4 deiodinase 2, acetyl-CoA carboxylase 1 and IRS-1, in liver and retroperitoneal adipose tissue of food-restricted rats allowed precise measures of their changes irrespective of the shrinking of the tissue, the loss of cells or changes in cell size, factors that deeply complicate the comparison between changing tissue conditions. The percentage results obtained with the present methods were essentially the same obtained with the delta-delta procedure and with individual cDNA standard curve quantitative RT-PCR estimation.

**Conclusion:**

The method presented allows the comparison (i.e. as copies of mRNA per cell) between different genes and tissues, establishing the degree of abundance of the different molecular species tested.

## Introduction

Real-time PCR analysis of gene expression is probably the method of choice for the establishment of hormone-, metabolite- or drug-induced modulation of the metabolic and hormonal milieu in most organs [1]. The results allow for comparison of the strength of replication (i.e. specific mRNAs abundance) of the genes/alleles under study between comparable groups of treated or experimental and control individuals. The comparisons are more often referred to "constitutive" genes, which are expected not to change under the experimental conditions because of their lack of reactivity, unrelatedness to the pathways studied or experimentally observed resilience to change. The results are usually presented as percentages of the controls or expressed in arbitrary units that allow for comparison, but seldom for quantitative analysis [2].

Quantitative PCR procedures are of limited application since they require considerable investments of time, resources and expertise [3,4].

The "comparative" approach is useful for a large number of experimental setups, and yields most of the data being currently published. A growing number of authors feel the need to include data on the number of complete PCR cycles necessary to achieve a given pre-established level of fluorescence in the analytical system used, but this is often additional information that just hints at the relative real abundance of experimental data. The "comparative" approach also requires that no additional changes in cellularity or tissue or organ mass occur, since then the comparisons with controls may be significantly skewed. The case of adipose tissue is paradigmatic of this situation: in the field of obesity many studies take this tissue as the subject of research, but its changes in size, cell count and cell size are seldom taken into account when comparing groups treated with powerful slimming agents and untreated (and unchanged) controls [5].

In this study we have developed a simple procedure for the quantification of specific mRNAs in relation to organ weight or cell numbers, so as to make fully comparable the data of controls and experimental subjects. Thus, we have studied the changes in the expression of a number of genes under the challenge of a 10-day period of limited food availability, a situation that is well within the physiological range and is akin to dietary energy restrictions in humans and induces significant changes in the expression of adipose tissue genes [6]. Decreased food energy reduces the mass of most adipose tissue locations in rats [7]. The study includes liver, i.e. an organ not expected to change too much under this limited dietary treatment and a location of white adipose tissue, which has been found to respond to decreased energy intake.

## Methods

### Animals and sample preparation

Adult male overweight Wistar rats [8], initially weighing 355 ± 5 g, and kept under standard conditions of housing and feeding were used. Two groups of 7 rats each were randomly selected: controls (C) and food-restricted (FR). The controls had free access to pellet food, and the FR were allowed only a 60% of the food consumed by C. FR rats completely ate the food allotted each day. On day 10, the rats were killed and the liver and retroperitoneal fat pads were excised, weighed, sampled, frozen and kept al -80°C.

The animals were handled and killed following the procedures approved by the University of Barcelona Animal Welfare and Ethics Committee in full compliment of the norms and procedures set forth by the European Union and the Governments of Spain and Catalonia.

### Nucleic acids measurement and tissue cellularity

Tissue samples were used for the estimation of total DNA, using a standard fluorimetric method with 3,5-diaminobenzoic acid (Sigma, St Louis MO USA) and bovine DNA (Sigma) as standard [9]. Tissue DNA content allowed the calculation of the number of cells per g of tissue and in the whole liver, based on the assumption that the DNA content per cell is constant in mammals; here we used the genomic DNA size data [10] for somatic rat cells (5.60 pg/cell). Total liver/adipose tissue cell numbers included not only hepatocytes/adipocytes, but immune system, endothelial and other minority types of cells as well.

Mean cell volume was estimated from the number of cells and the volume of the organ, calculated using a liver density of 1.10 g/mL and 0.90 for adipose tissue [11].

Total tissue RNA was extracted using the Tripure reagent (Roche Applied Science, Indianapolis IN USA), and were quantified in a ND-100 spectrophotometer (Nanodrop Technologies, Wilmington DE USA). RNA samples were reverse transcribed using the MMLV reverse transcriptase (Promega, Madison, WI USA) and oligo-dT primers.

Real-time PCR (RT-PCR) amplification was carried out using 10 μL amplification mixtures containing Power SYBR Green PCR Master Mix (Applied Biosystems, Foster City, CA USA), equivalent to 8 ng of reverse-transcribed RNA and 300 nM primers. Reactions were run on an ABI PRISM 7900 HT detection system (Applied Biosystems) using a fluorescent threshold manually set to OD 0.500 for all runs.

### Outline of the semiquantitative method for the measurement of gene expression

The quantitative measurement of mRNAs concentration in a given tissue requires to know: a) the efficiency of RNA extraction from the tissue; b) the percent effectiveness of the mRNA to cDNA retro-transcriptase process; c) the quantitative efficiency of the RT-PCR amplification; and d) the quantitative estimation of the number of transcripts generated in the RT-PCR process. A fully quantitative analysis would require the specific measurement of each of these four parameters for each transcript analyzed. However, procedure a, i.e. the effectiveness of RNA extraction has been repeatedly studied and found to be practically quantitative. Likewise, the efficiency of the real time PCR amplification step (point c) is very high [12], and has been followed using cDNA probes (see below). The main problems arise from points b and d. The latter has been estimated using the parameters of the fluorescence analysis system as described below. However, the critical point of the efficiency of the retro-transcriptase step (point b) could not be easily circumvented and is, probably the link of the calculation chain with lower efficiency. Oligo dT primers were used to enhance the representativeness of cDNAs from mixed mRNA populations [13]. The efficiency of the retro-transcriptase step has been linked to the length of the transcripts and also to the "noise" and abundance of other transcripts [14]. Few studies have tackled the problem, and give indications that range from 20% to 6% efficiency for normal or poorly represented transcripts when using a system similar to ours [14].

In our approximation to more quantitatively comparable data we tried to estimate and apply all the corrections available to the calculations except for the critical point of retro-transcriptase efficiency. We applied a flat 20% efficiency (based on ref. 14 data) to all calculations, which results in the estimations being only approximate and not quantitative. For this reason we consider that our estimations are "semiquantitative" and treat them as such.

The system of calculation we applied requires the estimation of the number of transcripts resulting in the final lecture of fluorescence of the system. Since the PCR procedure implies a duplication of the cDNA chains at each cycle, we obtain the relationship:

Tf = Ti·2^Z^

i.e. the number of final transcripts or copies Tf is dependent on the initial number of chains Ti and the number of duplication cycles Z. This equation is often [14] presented as:

Tf = Ti·(1+R)^Z^

where R is a factor that corrects for the eventual non-quantitative duplication of the initial transcripts because of possible alterations in the system. In the present study, in all measurements done, R was consistently equal to 1 – which means that the efficiency of the PCR step (point d) was quantitative and uniform for all samples-, thus we reverted to the simplified equation 1.

The system we used for real-time PCR established the number of cycles Z at which a given overall fluorescence is achieved. This set point is the same for all analyses and is preestablished in the instrument-based procedure. The linearity of the reaction is established by checking whether sequentially diluted cDNA samples result in proportionally increased numbers of cycles (in a log scale) [15]; we routinely included this check to ward off blank- or dilution-derived sources of error and to determine the sensitivity and efficiency of the amplification process.

The final (preset) fluorescence recorded by the system is proportional to the number of bases in the final reaction. We can thus rewrite equation 1:

Bf = Bi·2^Z^

where Bf is the final number of bases in the cDNAs and Bi is the initial amount of bases present in the cDNA population obtained from tissue mRNAs. Obviously,

Bi = Ti·L

where L is the length of the transcript, i.e. the number of bases between both extremes of the two probes for each gene. Similarly,

Bf = Tf·L

Since we assume that the fluorescence is proportional to the number of bases, the estimation of Bf (or Tf) must be done using external calibration curves (as explained below). In an experimental setup, the determination of the number of cycles necessary to achieve the preset fluorescence threshold (i.e. using standard chains of cDNA for the gene including both up and down sequences), will allow us to apply this standardized and quantified Bf value to all other transcripts [15]:

Bf = (Ti·L)·2^Z^

which allows for the estimation of the (initial) number of cDNA copies for the given gene in the sample of cDNA used (Ti).

Since we can directly correlate the amount of cDNA used in a reaction vessel to a given weight of the tissue after applying the corrections (measured, estimated or calculated) for the efficiency of the processes to obtain cDNAs, duplicate cycling of cDNA copies by the RT-PCR procedure, and estimation of the final number of copies of cDNA transcripts obtained, we can establish the number of copies of the mRNA per g of tissue or in the whole organ. These calculations allow us to present the concentration of each specific mRNA in molar units, or to express the value as the mean number of mRNA copies per cell simply by applying the Avogadro number (6.022 × 10^23 ^molecules per mol). Since a critical step is only an approximate (not measured) value (i.e. the efficiency of the reverse transcriptase step) we present these data as simple approximations to the real figures (semiquantitative approach).

### Establishment of the system basic parameters

The oligonucleotides used for the preparation of the external calibration curves were prepared from rat RNA. By using reverse transcriptase and oligo dT primers, cDNAs were obtained; they were amplified through the polymerase chain reaction, run on agarose gels and purified using the Hi-pure PCR Products Purification kit (Roche Applied Biosystems). Seven genes were used to establish the parameters of the system; the list, and the probes used can be seen in Table 1.

**Table 1:** Genes for cDNA standards and sequences of the primers used for their estimation.

Gene name	Gene	Direction	Sequence	Length
60S acidic ribosomal protein P0	*Arbp*	3' > 5'	GAGCCAGCGAAGCCACACT	62
		5' > 3'	GATCAGCCCGAAGGAGAAGG	
Cyclophilin A	*Ppia*	3' > 5'	CTGAGCACTGGGGAGAAAGGA	87
		5' > 3'	GAAGTCACCACCCTGGCACA	
Carbohydrate-responsive element-binding protein	*Wbscr14*	3' > 5'	TACTGTTCCCTGCCTGCTCTCC	116
		5' > 3'	ACTGCCCTTGTGGCTTGCTC	
Type II iodothyronine deiodinase	*Dio2*	3' > 5'	CGGTGGCTGACTTCCTGTTG	123
		5' > 3'	CACATCGGTCCTCTTGGTTCC	
Acetyl-CoA carboxylase 1	*Acaca*	3' > 5'	AGGAAGATGGTGTCCGCTCTG	145
		5' > 3'	GGGGAGATGTGCTGGGTCAT	
60S acidic ribosomal protein P0	*Arbp*	3' > 5'	CCCTTCTCCTTCGGGCTGAT	165
		5' > 3'	TGAGGCAACAGTCGGGTAGC	
Insulin receptor substrate 1	*Irs1*	3' > 5'	AATGAGGGCAGCTCCCCAAG	198
		5' > 3'	GGTCCTGGTTGTGAATCGTGAA	

Thus, the final number of copies per cell will be the product of the number of initial cDNA transcripts (Bi/L), the tissue mRNA yield (corrected by the efficiency of extraction and cDNA copying) and the number of cells (i.e. DNA vs. weight) in the same tissue.

### Comparison of the present method with standard procedures

The data obtained in the experiments described above were used to establish a direct standard comparison of expressions versus their corresponding controls using arbitrary units (delta-delta); the data were corrected by their relationships of mRNA abundance with respect to control constitutive genes (in this case cyclophilin and Arbp) [16].

A second -quantitative- approach was the comparison of the experimental data with standard calibration curves obtained using purified cDNAs at varying concentrations [13].

## Results and Discussion

The set of calculations presented here facilitates the final estimation of the actual concentration or presence (in absolute numbers) of the mRNA corresponding to the genes studied through RT-PCR. This small advancement in the usefulness of that procedure improves its yield by allowing the comparison between the strength of the expression of different genes, correcting for changes in organ size and cell size. This is specially important for studies on WAT [17], but -as the results show- also for other organs such as liver.

**Figure 1:**
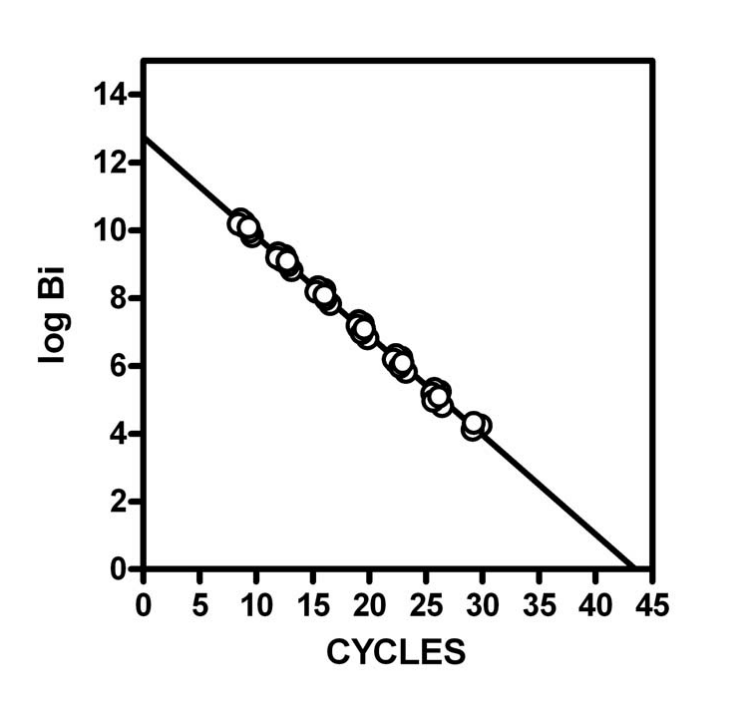
**Relationship between the number of RT-PCR cycles and the initial copies used (expressed as the log of the initial number of bases) in seven cDNA standards**. Parameters of the regression lines obtained for the individual (and combined) cDNAs are shown on Table 2. Each point of the line shown represents an individual measurement (N = 47). The most concentrated initial cDNA was 10^8^, and was the same for all the transcripts tested. This stock solution was successively diluted 1/10 to obtain the rest of concentrations, down to 10^2 ^copies of cDNA per tube.

Different initial amounts of these master genes were amplified through RT-PCR; Figure 1 shows the results obtained when plotting the number of bases found as a function of the number of cycles. In this case, the number of bases was derived from the known amount of cDNA standard for the specific gene used.

In Figure 1 (and the complementing Table 2), the equation for the regression line shows a slope close to -log 2 (deduced, but also found experimentally: -0.302 versus -0.293 ± 0.003, i.e. a mean 3% deviation over the theoretical figure). This formula coincides with a logarithmic expression of equation 3

**Table 2:** Parameters of the regression lines obtained for the individual (and combined) cDNAs shown on Figure 1

cDNA standards used	Slope	Log Bi (x = 0)	r^2^
60S acidic ribosomal protein P0 (62 bp)	-0.298 ± 0.001	12.71 ± 0.04	9998
Cyclophilin A	-0.307 ± 0.003	12.98 ± 0.06	9996
Carbohydrate-responsive element-binding protein	-0.296 ± 0.002	12.85 ± 0.03	9999
Type II iodothyronine deiodinase	-0.296 ± 0.002	12.73 ± 0.01	10000
Acetyl-CoA carboxylase 1	-0.292 ± 0.002	12.65 ± 0.04	9998
60S acidic ribosomal protein P0 (165 bp)	-0.291 ± 0.001	12.88 ± 0.03	9999
Insulin receptor substrate 1	-0.291 ± 0.001	12.81 ± 0.03	9999
**All data combined **(line represented)	-**0.293 ± 0.003**	**12.75 ± 0.06**	**9960**

logBi = -log2·Z + logBf

**Figure 2:**
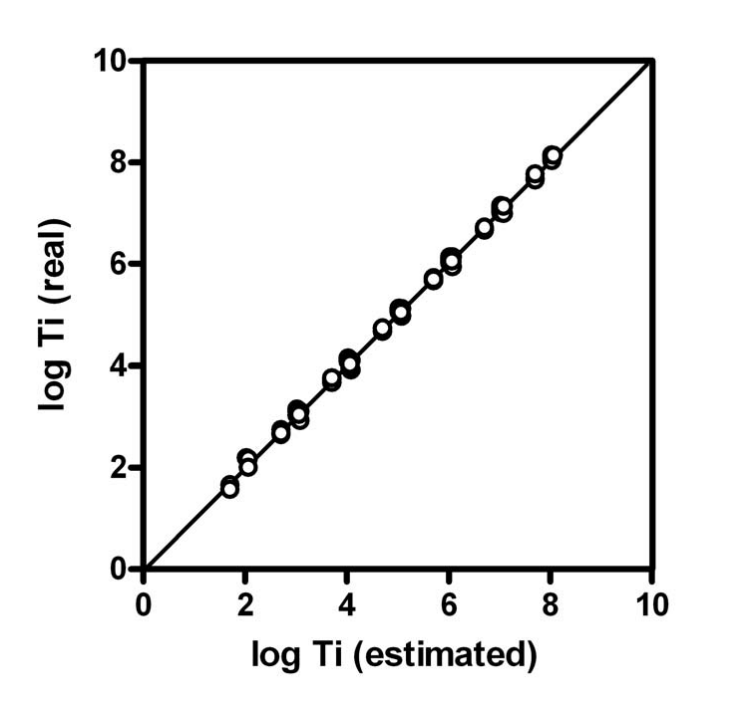
**Relationship between the number of added cDNA standard transcripts and those experimentally found**. The cDNA transcripts used were those listed in Figure 1. Each point represents an individual measurement (N = 87). Slope = 1.006 ± 0.004; Y intercept = -0.038 ± 0.021; r^2 ^= 0.9987.

We have found experimentally that the genes tested respond to equation 7 with a very high degree of correlation between expected and obtained values by using the cDNAs standards (Figure 2). The resulting line corresponds to the equation:

logTi_real _= 1.01·logTi_estimated _- 0.04

that established a practical identity between both estimated and real Ti values (r^2 ^= 0.999), and confirms the applicability of the procedure to a number of different genes of different variability and abundance, and also using transcripts of different sizes.

Table 3 shows the weight and cellularity of the tissues used. As expected, the number of cells in liver was practically unchanged with limited feeding, but cell size shrunk by about 35% (in the same proportion as the liver weight). The differences in tissue weight and cell size can be directly traced to the loss of energy substrates, such as glycogen (and water) [18] and lipids [19].

**Table 3:** Liver and adipose tissue cellularity

Parameter	Organ	Units	Control	Food-restricted
Tissue weight	liver	g	11.3 ± 0.4	7.4 ± 0.2 *
	WAT		9.07 ± 0.92	5.01 ± 0.67*
Number of cells	liver	× 10^9^	4.44 ± 0.26	4.31 ± 0.18
	WAT		0.37 ± 0.02	0.29 ± 0.03*
Mean cell size	liver	pL	2.42 ± 0.11	1.55 ± 0.07 *
	WAT		25.7 ± 2.0	17.5 ± 2,6 *

The data are the mean ± sem of 7 different animals. Differences between groups: * = P < 0.05 versus controls.

In retroperitoneal WAT we observed a reduction in organ size (almost by half) due to both a decrease in the number of cells it contained (loss of about 22% of the cells) and decreased cell size (by about 32%). The decrease in mean cell size and cell numbers do not fully add to the tissue loss of WAT weight because with its mass shrinkage, largely due to the loss of fat [17] which affects its density. In addition their large mean size (in the range of ten-fold the mean size of liver cells) could not fully correspond to adipocytes, the prevailing cell type, because a variable number of other smaller cell types, such as preadipocytes, macrophages, and stem cells [20] coexist with adipocytes and their proportion is altered by obesity, stress and other conditions [21]. In any case, the data presented are a fairly valid approximation because of both the large predominance of hepatocytes in liver and adipocytes in WAT. In addition, the results, when presented per unit of cell-DNA (i.e. copies per cell of the mRNAs) fully correspond to the reality of the tissue irrespective of the type of cell that mostly contains the mRNA copies of the gene studied.

The loss of energy (mainly lipid) and loss of cells in WAT are in agreement with the supply of lipids to the liver under conditions of energy scarcity [22], resulting in the progressive mobilization of its triacylglycerol droplets and the corresponding diminution of its storage of circulating energy substrates.

Comparison of the data obtained using the present approach and those from the delta-delta or quantitative RT-PCR using standard cDNA standard curves can be seen in Table 4. The use of individual cDNA standards for the genes selected gave closely similar results to ours using a single generic common standard curve, and were also similar (when expressed as percentages) to those of the delta-delta procedure. The overall coincidence of results using genes of different abundance from different tissues supports the validity of our approach. The advantage gained by using the procedure we postulate here is, thus, that there is no need of individual specific cDNA calibration curves for each gene (the fluorescence-base pairs curve is sufficient) and, especially, that using our approach different tissues and different genes (i.e. of varying abundance) can be easily compared and conclusions as of their abundance can be drawn.

**Table 4:** Comparison of results obtained using a delta-delta (after correction using constitutive genes) method, quantitative RT-PCR approach (individual cDNA standard curves) and our postulated method (common cDNA bp-based standard curve) on liver and WAT from animals with restricted access to food and their controls.

ORGAN and gene	Group	Delta-delta	Individual RT-PCR	Present method
LIVER				
Carbohydrate-responsive element-binding protein	Control	100 ± 4	100 ± 5	100 ± 5
	Restricted	76 ± 4	79 ± 4	79 ± 4
Acetyl-CoA carboxylase 1	Control	100 ± 8	100 ± 3	100 ± 3
	Restricted	50 ± 12	47 ± 21	46 ± 1
Insulin receptor substrate 1	Control	100 ± 8	100 ± 9	100 ± 8
	Restricted	116 ± 10	115 ± 10	115 ± 10
ADIPOSE TISSUE				
Carbohydrate-responsive element-binding protein	Control	100 ± 12	100 ± 10	100 ± 9
	Restricted	24 ± 4	27 ± 5	27 ± 5
Type II iodothyronine deiodinase	Control	100 ± 6	100 ± 6	100 ± 5
	Restricted	45 ± 3	46 ± 2	46 ± 2
Acetyl-CoA carboxylase 1	Control	100 ± 8	100 ± 13	100 ± 11
	Restricted	55 ± 14	53 ± 14	53 ± 11
Insulin receptor substrate 1	Control	100 ± 13	100 ± 12	100 ± 11
	Restricted	28 ± 5	29 ± 5	28 ± 4

The data are the mean ± sem of 7 different animals and are expressed in all the cases as the percentage of the mean controls' values for easier comparison between the methods using homologous units. The base data used for comparisons in the case of the postulated method (and the individual quantitative RT-PCR method) was fmol/unit of total tissue RNA weight.

In Table 5 we can see the application of the semiquantitative methodology proposed. Many of the genes tested gave very similar numbers of cycles in their RT-PCR estimation for both controls and food-restricted animals, in spite of small differences meaning large changes in specific mRNA abundance because of the logarithmic scale. If the only correction applied is the control of charge (comparison with constitutive gene expression), then the results obtained will miss any modification due to changes in cell size because of water, glycogen or fat content variation. In addition, the simple correction for RNA charge and constitutive gene expression does not take into account the overall effect of the selected gene expression for the whole organism because of changing size and cellularity of the organ studied.

**Table 5:** Mean number of copies per cell of the mRNAs for the selected genes in liver and adipose tissue of overweight male rats subjected to food restriction

Gene name	Control		Restricted feeding	
	
	Cycles	cpc	Cycles	cpc
	Liver			
60S acidic ribosomal protein P0 (62 bp)	20.1 ± 0.1	1157 ± 50	20.1 ± 0.1	801 ± 38*
Cyclophilin A	19.4 ± 0.1	1261 ± 44	19.6 ± 0.1	855 ± 13*
Carbohydrate-responsive element-binding protein	22.8 ± 0.1	94 ± 6	23.3 ± 0.1*	54 ± 3*
Acetyl-CoA carboxylase 1	23.4 ± 0.2	50 ± 3	24.6 ± 0.1*	19 ± 1*
Insulin receptor substrate 1	25.0 ± 0.2	13 ± 1	24.9 ± 0.2	12 ± 1
	Retroperitoneal WAT			
60S acidic ribosomal protein P0 (62 bp)	19.7 ± 0.1	160 ± 18	19.7 ± 0.1	191 ± 29
Cyclophilin A	19.1 ± 0.1	193 ± 30	19.4 ± 0.1	149 ± 7
Carbohydrate-responsive element-binding protein	24.4 ± 0.3	4.3 ± 0.6	26.2 ± 0.4*	1.0 ± 0.2*
Type II iodothyronine deiodinase	27.0 ± 0.1	0.63 ± 0.10	28.1 ± 0.2*	0.29 ± 0.03*
Acetyl-CoA carboxylase 1	23.0 ± 0.2	7.6 ± 1.8	23.5 ± 0.6	6.8 ± 2.2
Insulin receptor substrate 1	26.6 ± 0.2	0.46 ± 0.05	27.9 ± 0.4*	0.15 ± 0.04*

The data are the mean ± sem of 7 different animals; cpc = copies of the corresponding mRNA per cell. Statistical significance of the differences between groups (Student's t test) * = P < 0.05.

When the additional corrections proposed are applied, even taking into account the relative individual imprecision of the effectiveness of the transcription process, we can give an estimate of the relative importance of the expression by relating the results to individual cells. This approach circumvents the problems posed by changes in tissue mass due to cell size changes, and can be further corrected by cell number changes for a better understanding of the physiological consequences of the gene expression analysis. The application of this copies-per-cell approach allows also for the relative comparison of the expression of different genes. Thus, we observe that the number of copies of the 60S acidic ribosomal protein is not altered in adipose tissue, but is down by a third in liver as a consequence of restricted feeding. A similar situation occurs with cyclophilin A; since both are commonly used "constitutive reference genes". In spite of the proven use of these genes for the control of RNA charge, the existence of differences in treated versus control groups when expressed as copies per cell cast doubts on their assumed unchanged physiological function.

The expression in liver of insulin receptor substrate 1 yields a small number of copies, but it is not changed by food restriction, whereas the more abundant acetyl-CoA carboxylase gene expression is strongly reduced by food restriction, a logical change under the limited availability of lipogenic substrates. The copies-per-cell approach also allows for comparison between different tissues, thus we can observe that the number of copies per cell of the mRNAs for all the genes presented in Table 4 for WAT are lower than those of the much more active liver cells. In WAT, cyclophilin and 60S acidic ribosomal protein changes were small (not significant) in spite of a marked reduction in cell size (a behavior different from that of liver for these same genes). The expression of carbohydrate-responsive element-binding protein and insulin receptor substrate 1 decreased to about 20% in the food-restricted group, in agreement with the "wasting mode" adopted by the tissue under the ordeal of insufficient dietary fuels. A similar situation is observed in the T4-deiodinase, which main function is exacerbate thyroid hormone effects, akin to energy wasting [23]; under the strict conservation scheme of energy preservation, thyroid function is depressed [24] in part by decreasing peripheral T4 to T3 conversion. WAT acetyl-CoA carboxylase expression was unchanged, suggesting a functionally active lipogenic pathway that may help to control the loss of energy endured by the tissue.

In the cases of a number of regulatory proteins of a tissue with limited metabolic activity as is the WAT, the copies per cell of their mRNAs may be very low, with values lower than a single copy per cell. This scarcity can only be interpreted as the combination of both a) the limited need for these particular proteins synthesis such as that related to hormone path signalling, and b) that not all the cells in the tissue contain the pathway or express the corresponding gene. The relative heterogeneity of WAT cellular populations [20] fits this interpretation: a few cells may have a sizeable number of copies of the mRNAs and most other have none; the postulated approach favours the identification of such cases, which may be easily passed over when the data are simply presented as a percentage of their controls.

In any case, the approach we postulate widens the possibility of physiological interpretation through comparison of the regulatory processes based on gene expression by extending the comparisons to other cells and genes and introducing a relative measure of quantitative importance of the specific gene expression studied.

## Competing interests

The author(s) declare that they have no competing interests.

## Authors' contributions

MMR did most of the experimental work; MMG and ME realized partial experiments and devised solutions to a number of experimental problems; JAFL established the numerical relationships and did all the computer work; MA designed the experiment and wrote the paper. All Authors participated in the design and final edition of the manuscript, as well as on the streamlining of the procedures and its direct application to experimental situations.
